# Natural or Naturalized? Phylogeography Suggests That the Abundant Sea Urchin *Arbacia lixula* Is a Recent Colonizer of the Mediterranean

**DOI:** 10.1371/journal.pone.0045067

**Published:** 2012-09-17

**Authors:** Owen S. Wangensteen, Xavier Turon, Rocío Pérez-Portela, Creu Palacín

**Affiliations:** 1 Department of Animal Biology, University of Barcelona, Barcelona, Spain; 2 Center for Advanced Studies of Blanes (CEAB-CSIC), Blanes (Girona), Spain; University of Gothenburg, Sweden

## Abstract

We present the global phylogeography of the black sea urchin *Arbacia lixula*, an amphi-Atlantic echinoid with potential to strongly impact shallow rocky ecosystems. Sequences of the mitochondrial cytochrome *c* oxidase gene of 604 specimens from 24 localities were obtained, covering most of the distribution area of the species, including the Mediterranean and both shores of the Atlantic. Genetic diversity measures, phylogeographic patterns, demographic parameters and population differentiation were analysed. We found high haplotype diversity but relatively low nucleotide diversity, with 176 haplotypes grouped within three haplogroups: one is shared between Eastern Atlantic (including Mediterranean) and Brazilian populations, the second is found in Eastern Atlantic and the Mediterranean and the third is exclusively from Brazil. Significant genetic differentiation was found between Brazilian, Eastern Atlantic and Mediterranean regions, but no differentiation was found among Mediterranean sub-basins or among Eastern Atlantic sub-regions. The star-shaped topology of the haplotype network and the unimodal mismatch distributions of Mediterranean and Eastern Atlantic samples suggest that these populations have suffered very recent demographic expansions. These expansions could be dated 94–205 kya in the Mediterranean, and 31–67 kya in the Eastern Atlantic. In contrast, Brazilian populations did not show any signature of population expansion. Our results indicate that all populations of *A. lixula* constitute a single species. The Brazilian populations probably diverged from an Eastern Atlantic stock. The present-day genetic structure of the species in Eastern Atlantic and the Mediterranean is shaped by very recent demographic processes. Our results support the view (backed by the lack of fossil record) that *A. lixula* is a recent thermophilous colonizer which spread throughout the Mediterranean during a warm period of the Pleistocene, probably during the last interglacial. Implications for the possible future impact of *A. lixula* on shallow Mediterranean ecosystems in the context of global warming trends must be considered.

## Introduction

The European black sea urchin *Arbacia lixula* (Linnaeus, 1758) is currently one of the most abundant echinoids in shallow rocky habitats of the Mediterranean [1], where it has the potential to greatly influence benthic communities with their grazing activity [2]–[4]. *A. lixula* has a considerable trophic plasticity, ranging from omnivory to strict carnivory [5] and its scraping predatory behaviour can bulldoze the substrate bare of erect and encrusting algae and sessile animals. *A. lixula* broadly overlaps its habitat with the common edible sea urchin *Paracentrotus lividus* (Lamarck, 1816). Both species are traditionally thought to have the ability to trigger the development of subtidal barren zones of reduced benthic productivity and diversity [6]–[9]. However, new and increasing evidence suggests that *A. lixula* could actually be playing the principal role in producing and maintaining these barrens [10] and that this trend could be worsening in the near future due to foreseeable climatic changes [11].

*Arbacia lixula* is commonly regarded as a typical native species in the Mediterranean fauna [12], since it is currently found in shallow rocky shores all along the Mediterranean, often at high densities, and has been so since historical times. However, its tropical affinities have been suggested for a long time. Based on the lack of Mediterranean fossil record, Stefanini [13] and Mortensen [14] stated that *A. lixula* (reported as *A. pustulosa*), probably originated at the Tropical Atlantic region, from where it spread into the Mediterranean. Kempf [15], Tortonese [16] and Fenaux [17] also considered that *A. lixula* was a thermophilous species.

In NW Mediterranean, increasing abundances over time have been reported for this species. In 1950, Petit *et al*. reported that *Arbacia lixula* had become abundant in Marseilles during the previous 30 years [18], despite Marion had described it as rare in the same area in 1883 [19]. More recently, Francour *et al.* reported a 12–fold increase in the abundance of *A. lixula* in Corsica over a period of nine years (1983–1992) and speculated that a long term rise in the water temperature could have been the cause for this proliferation [20]. In the same period (1982 to 1995), a 5-fold increase in *A. lixula* densities was reported at the Port-Cros Marine Reserve (France) [21]. On the other hand, in a recent 5-year follow-up (2003–2008) at Ustica Island (Southern Thyrrenian Basin), a positive correlation was found between the gonado-somatic index of adult *A. lixula* and summer surface water temperature, suggesting increased reproductive potential with temperature [10].

*Arbacia* is an ancient genus with a fossil record that dates back to the Paleocene [22] whose distribution is mainly Neotropical. Unlike other sea urchin genera, *Arbacia* has a history of latitudinal shifts [23], and the five extant species inhabit mainly temperate and tropical shallow waters [24], being mostly allopatric. Only one species, *A. dufresnii*, is able to live in cold Subantarctic waters. *A. lixula* is the only species in the genus that lives in the Old World. Its present distribution includes Brazil, the African Atlantic coast from Morocco to Angola, the East Atlantic archipelagos of Cape Verde, Canaries, Madeira and Azores, and the whole Mediterranean basin, excluding the Black Sea. It has never been reported from the Atlantic European coast north of Gibraltar (J. Cristobo, X. Troncoso, N. V. Rodrigues; pers. comms.), probably due to the low sea surface temperature originated by the southward Portugal Current [25].

Recently, Lessios *et al.* [26] presented an exhaustive phylogenetic study of genus *Arbacia,* using sequences of the mitochondrial COI (cytochrome *c* oxidase I) and the nuclear gamete recognition protein bindin, which has clarified many interesting questions on inter-specific relationships within this remarkable genus. Notably, the sequence of speciation events was consistently reconstructed and their divergence times were reliably estimated. Thus, the splitting between *A. lixula* and its sister species, the NW Atlantic *A. punctulata,* was estimated to have taken place some 2.2–3.0 Mya (millions years ago) based on COI sequences, or 1.9–3.3 Mya based on bindin sequences. The phylogeny of bindin sequences also allowed these authors to infer that Brazil populations separated from the rest of *A. lixula* some 1.8–3.4 Mya; i.e. very early in the evolution of this species (however, only 5 individuals from Brazil were used in the analysis, and no estimation could be inferred for the same event from mitochondrial sequences, due to the unresolved position of the Brazilian clade within other *A. lixula* haplotypes).

Yet, many questions remain open about the intra-specific relationships of *Arbacia lixula*. Considering its unusually wide present distribution area, which ranges from equatorial waters to temperate Mediterranean, the great colonizing potential shown by this species, including the ability to cross trans-oceanic barriers to gene flow [26], and the massive potential impact of its behaviour on coastal ecosystems, further research on its phylogeography and population genetics is necessary in order to elucidate the history and ongoing processes that shape the distribution of the species. In this work, we present a phylogeographic study using the mitochondrial marker COI, based on a representative sample of individuals covering most of the distribution area of *Arbacia lixula.* Our goals were to answer relevant questions concerning the history and present-day distribution of the species: What are the relationships between the main geographic areas where the species is found? Do the main geographic barriers to gene flow, that are known to regulate the genetic structure of many other marine organisms, affect the present-day genetic structure of this species? Can recent geographic and/or population expansion events be traced and reconstructed by analysing the signature left in sequence data of this species?

## Methods

### Ethics Statement

Field sampling required for this work involved only invertebrate species which are neither endangered nor protected. All necessary permits for sampling at localities placed inside protected areas (Cabrera National Park, Columbretes Islands Marine Reserve & Scandola Nature Reserve) were previously obtained from the competent authorities. Non-destructive sampling techniques (external soft tissue biopsy) were used in these localities in order to minimize impact on the ecosystems.

### Sampling

**Figure 1:**
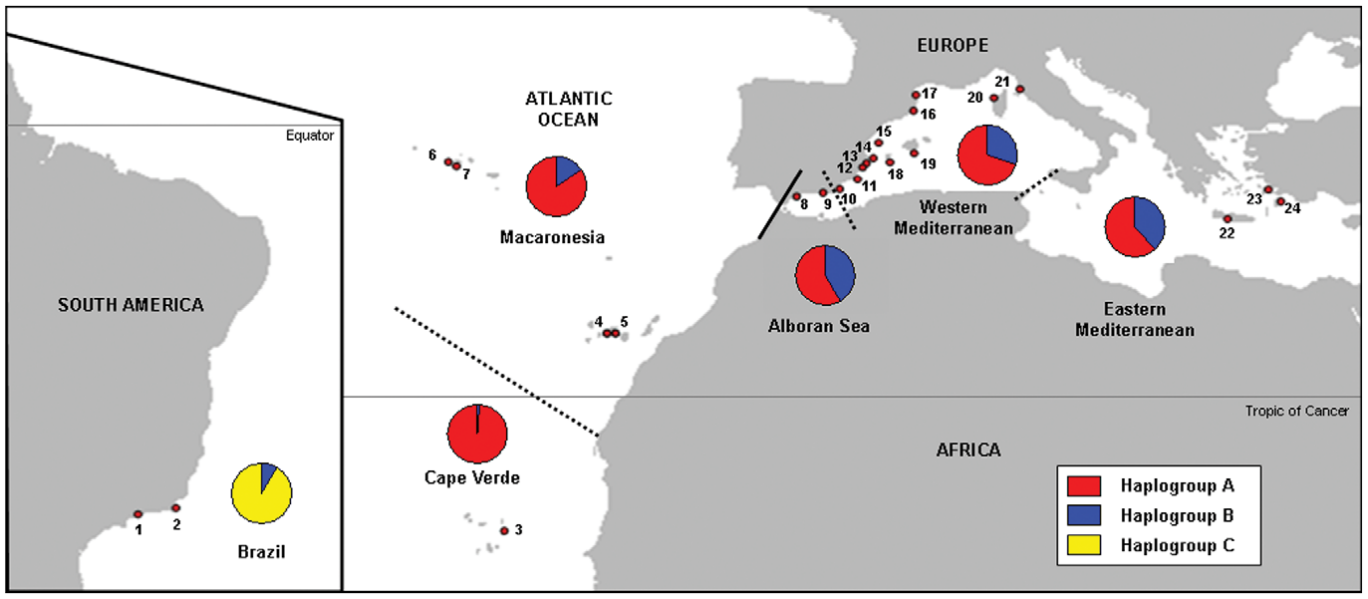
Sampling localities for *Arbacia lixula* populations. See Table 1 for locality names and coordinates. Borders between regions are indicated by solid bold lines and borders between sub-regions are represented by dotted lines. Pie charts of haplogroup frequencies are shown for the six sub-regions in which the three studied regions have been subdivided.

Between April 2009 and July 2011, we obtained samples from 24 localities belonging to three predefined regions: West Atlantic, East Atlantic and Mediterranean (see Fig. 1 and Table 1). For more detailed analyses, we further subdivided the East Atlantic region in two sub-regions (Cape Verde and Macaronesia), while the Mediterranean was divided in three sub-basins (Alboran Sea, West Mediterranean and East Mediterranean). The sampled localities were: two from Brazil, one from Cape Verde, four from Macaronesian archipelagos, two from the Alboran Sea, twelve from West Mediterranean and three from East Mediterranean. 15 to 30 adult *Arbacia lixula* individuals (average: 25.2) per location were sampled. In all cases, tissue samples were stored in absolute ethanol at −20°C until processed.

**Table 1:** *Arbacia lixula.* Sampling localities.

Label	Locality	Code	Region	Sub-region	Latitude/Longitude
1	Itaipu	ITA	W. Atlantic	Brazil	−22.974910/−43.050456
2	Cabo Frio	CFR	W. Atlantic	Brazil	−22.890409/−41.998186
3	Boavista	BOA	E. Atlantic	Cape Verde	16.136858/−22.941055
4	Los Gigantes	GIG	E. Atlantic	Macaronesia	28.200925/−16.8294084
5	Tenerife (East)	TEN	E. Atlantic	Macaronesia	28.100823/−16.478088
6	Faial	FAI	E. Atlantic	Macaronesia	38.522720/−28.620937
7	Pico	PIC	E. Atlantic	Macaronesia	38.423336/−28.415823
8	Torremuelle	TOR	Mediterranean	Alboran Sea	36.577369/−4.565396
9	La Herradura	HER	Mediterranean	Alboran Sea	36.721044/−3.728487
10	Carboneras	CAR	Mediterranean	W. Medit.	36.993869/−1.890274
11	Palos	PAL	Mediterranean	W. Medit.	37.634580/−0.693749
12	Villajoyosa	VIL	Mediterranean	W. Medit.	38.509007/−0.212885
13	Benidorm	BEN	Mediterranean	W. Medit.	38.502530/−0.128329
14	Xabia	XAB	Mediterranean	W. Medit.	38.752880/0.224511
15	Columbretes	CLM	Mediterranean	W. Medit.	39.898115/0.685179
16	Tossa	TOS	Mediterranean	W. Medit.	41.722109/2.939914
17	Colera	COL	Mediterranean	W. Medit.	42.391077/3.155390
18	Formentera	FOR	Mediterranean	W. Medit.	38.693415/1.376867
19	Cabrera	CAB	Mediterranean	W. Medit.	39.155689/2.944236
20	Scandola	SCA	Mediterranean	W. Medit.	42.361842/8.549023
21	Populonia	POP	Mediterranean	W. Medit.	42.993752/10.498702
22	Crete	CRE	Mediterranean	E. Medit.	35.171626/24.400875
23	Kos	KOS	Mediterranean	E. Medit.	36.888477/27.308822
24	Rhodes	ROD	Mediterranean	E. Medit.	36.319364/28.207868

### DNA Amplification and Sequencing

Total DNA was extracted using REDExtract-N-Amp Tissue kit (Sigma–Aldrich, www.sigma.com) from either one tube foot or a tiny portion (5–10 mg) of gonad. A fragment of the COI gene was amplified and sequenced using specific primers designed using the complete genome sequence of *A. lixula* mitochondrion [27] with primer 3.0 [28], as follows: COIARB-F: 5′-TTC TCT GCT TCA AGA TGA C-3′, COIARB-R: 5′-CTA TAA TCA TAG TCG CTG CT-3′, COIAL-R: 5′-GCT CGG GTA TCT AGG TCC AT-3′. Most individuals were amplified using the COIARB-F/COIARB-R pair, but some individuals belonging to Atlantic populations had to be amplified using COIARB-F/COIAL-R instead. PCR amplification reactions were performed in a 20 µl total-reaction volume with 10 µl of REDExtract-N-Amp PCR reaction mix (Sigma–Aldrich), 0.8 µl of each primer (10 µM), 6.4 µl of ultrapure water (Sigma–Aldrich) and 2 µl of template DNA. A single denaturing step at 94°C for 5 min was followed by 40 cycles (denaturation at 94°C for 40 s, annealing at 43°C for 45 s and extension at 72°C for 45 s) and a final extension at 72°C for 5 min in a S1000 dual thermal cycler (BioRad, www.bio-rad.com). The PCR products were purified and both strands sequenced in Macrogen (www.macrogen.com) using the same primers for the sequencing reaction.

### Genetic Diversity Analyses

All the sequences were edited in Bioedit [29] and aligned using ClustalW as implemented in Mega 5 [30]. The single nucleotide mutations found were double-checked by contrasting the agreement and quality of forward and reverse sequencing chromatograms. The Nei & Gojobori procedure with the Jukes & Cantor correction [31]–[32] implemented in Mega 5 was used for detecting positive natural selection. Sequences of the haplotypes found have been deposited in GenBank (accession numbers from JQ745096 to JQ745256).

Number of haplotypes (N_h_), haplotype diversity (H_d_) and nucleotide diversity (π) were computed with DnaSP v. 5.10 [33]. Haplotype richness was calculated with contrib v. 1.02 [34] using a rarefaction size equal to the smallest sample size (n = 15) and Student’s *t*-test was used for comparing its values between regions having more than two sampled locations (i.e., Eastern Atlantic and Mediterranean).

We used baps v. 5.2 (Bayesian Analysis of Population Structure) [35]–[36] for clustering the sampled haplotypes into monophyletic clusters of haplotypes (haplogroups). We ran five replicates for every value of the maximum number of clusters (k) up to k = 10. Haplotypes were assigned to one of the clusters by admixture analysis, performing 50 simulations from posterior haplotype frequencies. The assigned haplotype names reflect the haplogroup they belong.

### Phylogeography and Phylogeny

Relationships and geographical distribution of the haplotypes were analysed in a haplotype network constructed with network v. 4.6.0.0 (http://www.fluxus-engineering.com/sharenet.htm), which implements the median-joining method, in the absence of recombination [37]. The network was optimized using maximum parsimony criterion and the obtained loops were solved using criteria derived from coalescent theory [38]–[39]. In order to determine the putative ancestral haplotypes, the outgroup weights based on haplotype frequency and connectivity [40] were calculated for each haplotype using the tcs v. 1.21 program [41].

For phylogenetic analysis of the haplotypes obtained, we included a sequence of *Strongylocentrotus purpuratus* from GenBank (Acc. number NC_001453 [42]). Though the use of an outgroup sequence for rooting intraspecific genealogies has been shown to have little resolution [43], we nevertheless used it since the resulting tree is coherent with the outgroup weights calculated using tcs. We used jModeltest v. 0.1.1 [44], based on a hierarchical series of likelihood ratio tests [45] and the Bayesian Information Criterion (BIC), to assess the most appropriate nucleotide substitution model for our data. This condition was satisfied by the Tamura & Nei model [46] with a gamma correction (α = 0.240) (TrN + G). This evolution model was fed into MrBayes software v. 3.1.2 [47] and the haplotype tree was estimated under the BIC after 1 million generations of 8 MCMC chains with a sample frequency of 100 (10,000 final trees). After verifying that stationarity had been reached, the first 2,000 trees were discarded, an independent majority-rule consensus tree was generated from the remaining (8,000 trees), and it was drawn using mesquite v. 2.75 [48].

### Population Structure Analyses

Pairwise genetic distances between populations (*F_st_*) were calculated with arlequin v. 3.1 [49] considering the genetic distance between haplotypes, and their significances were tested by performing 40,000 permutations. The level of significance for these multiple tests was corrected by applying the B–Y false discovery rate (FRD) procedure [50]–[51]. Kruskal’s non-metric multidimensional scaling (MDS [52]) of *F*_st_ values was performed with RStudio [53] to graphically visualise these results. In order to have a different differentiation measure based only on haplotype frequencies, Jost’s *D* [54] was calculated using spade [55]. Negative values for *D* were corrected to zero. We calculated a confidence interval around the obtained values by 1,000 bootstrap replicates. We set this confidence interval, using the normal approximation, at the appropriate *P*-value following the B-Y correction as explained above. Significant differentiation was inferred when this confidence interval excluded zero.

Analyses of molecular variance (AMOVA) were performed to assess population structure, using conventional *F*-statistics (i.e. only with haplotype frequencies), and their significances were tested running 90,000 permutations in arlequin [56]. AMOVAs were performed using different population sets in order to test the significance of population structure among regions, or among sub-basins within regions. These AMOVAs were repeated also considering genetic distances between haplotypes, in order to check the robustness of the results.

The effect of isolation by geographical distance was assessed, for the whole dataset or separately for different populations sets, by the correlation of linearized genetic distances (*F*_st_/1–*F*_st_) [57] with geographical distances between localities. Though ideally the oceanic current patterns should be included in the geographical distances calculation, currently we do not know of any reliable method for accurately quantifying this, so we used the shortest distance by sea on Google Earth 6 (http://www.google.com/earth). The significance of the correlation was tested by the Mantel test procedure [58], implemented in arlequin, with 20,000 permutations for each analysis.

### Demographic History Inference

Demographic history was inferred for the three studied regions and for each sub-basin by analysing the mismatch distributions. Populations that have recently experienced a sudden demographic growth show unimodal distributions, whereas those at demographic equilibrium show multimodal distributions [59]. The expected mismatch distributions under a sudden expansion model were computed in arlequin using Monte Carlo simulations with 10,000 random samples. The sum of squared deviations (SSD) between observed and expected distributions was used as a measure of fit, and the probability of obtaining a simulated SSD greater than or equal to the expected was computed by randomisation. If this probability was >0.05, the expansion model was accepted, and its parameters θ_0_, θ_1_ and τ were calculated. For those populations showing large values for the final effective population size θ_1_, this method does not usually converge and flawed results could be obtained. In this case, we kept the value of τ calculated by this method, which is consistently robust [60], and used DnaSP to calculate the value of θ_0_ which minimized the SSD, letting θ_1_ have an arbitrary large value of 1000 [61]. In the case that the mismatch distribution was not unimodal, the data were fitted to a constant population size model [62]–[63] for graphical representation.

To estimate the approximate time of a demographic expansion (t) from coalescence methods, the relationship τ = 2 µkt was used [59] where τ is the mode of the mismatch distribution, μ is the mutation rate per nucleotide and k is the number of nucleotides of the analysed fragment. A range of mutation rates from 1.6% to 3.5% per million years was used for the COI gene, as calculated previously for echinoids [64]–[65].

In order to add more statistical support for population expansions, Tajima’s *D* test of neutrality [66], Fu’s *F*_s_ [67], and Ramos-Onsins & Rozas’ *R*_2_ [68] indices of population expansion were calculated using DnaSP. The confidence limits of Tajima’s *D* were obtained assuming that it follows the beta distribution [66], while statistical tests and confidence intervals for *F*_s_ and *R*_2_ were based on a coalescent simulation algorithm implemented in DnaSP, with 20,000 simulations. Harpending’s raggedness index *r* [69] was calculated using arlequin and its significance was tested using parametric bootstrapping (10,000 replicates). These indices were calculated for the three regions and the six predefined sub-regions.

## Results

### Genetic Diversity

We sequenced 635 bp of the mitochondrial gene COI from 604 *Arbacia lixula* individuals from 24 localities (Fig. 1 and Table 1). We found 135 polymorphic sites (21%), with a total of 144 mutations. All differences between haplotypes were substitutions, 42 of which were non-synonymous. The Nei-Gojobori Z-test did not detect any significant positive selection (*P*>0.95). A total of 161 haplotypes were obtained from all the sequences (Table S1). Of them, 126 (78.3%) were private haplotypes (found in only one locality) and 117 (72.7%) were represented by only one sampled individual. The number of haplotypes per locality ranged between 4 and 18. Haplotype diversity (H_d_) and nucleotide diversity (π) calculated for the whole geographical range were 0.912 (±0.007 SD) and 0.00658 (±0.00026 SD), respectively (Table 2). All diversity measures were remarkably uniform among localities within each East Atlantic or Mediterranean regions, but were quite different in the case of the two sampled localities in Brazil, having the smallest values in Itaipu (the westernmost and southernmost locality in our study). The haplotype richness in the Eastern Atlantic samples was higher than in the Mediterranean (*t* = 3.336, 20 d.f.; *P* = 0.0033), indicating that the Eastern Atlantic populations are more genetically diverse than their Mediterranean counterparts. The small number of samples available from Brazil prevented us from performing any diversity comparison of this area with other regions.

**Table 2:** *Arbacia lixula.* Estimates of genetic diversity for all locations and regions sampled.

Locality or region	*N*	*N_h_ (N_priv_)*	*r_hap_*	*H* ± *SD*	*π* ± *SD*
Itaipu	20	4 (3)	3.491	0.432±0.126	0.00074±0.00024
Cabo Frio	15	8 (7)	8.000	0.790±0.105	0.00594±0.00156
**Total W. Atlantic**	**35**	**11 (11)**	**5.935**	**0.605±0.096**	**0.00317±0.00098**
Boavista	27	15 (10)	10.172	0.920±0.038	0.00358±0.00067
Los Gigantes	24	12 (5)	8.698	0.851±0.064	0.00389±0.00092
Tenerife (East)	24	18 (10)	11.869	0.942±0.040	0.00577±0.00089
Faial	24	15 (7)	10.572	0.928±0.039	0.00444±0.00095
Pico	24	14 (5)	10.299	0.938±0.028	0.00528±0.00069
**Total E. Atlantic**	**123**	**56 (41)**	**10.924**	**0.921±0.019**	**0.00461±0.00040**
Torremuelle	27	14 (5)	8.638	0.826±0.069	0.00480±0.00065
La Herradura	26	15 (6)	9.999	0.917±0.037	0.00517±0.00040
Carboneras	26	15 (6)	9.750	0.905±0.041	0.00451±0.00051
Palos	28	12 (5)	8.031	0.860±0.047	0.00530±0.00062
Villajoyosa	30	16 (5)	9.596	0.894±0.044	0.00542±0.00058
Benidorm	29	12 (4)	7.808	0.842±0.051	0.00410±0.00033
Xabia	27	15 (5)	10.028	0.917±0.038	0.00544±0.00051
Columbretes	25	13 (7)	8.943	0.887±0.045	0.00549±0.00068
Tossa	29	15 (5)	8.980	0.877±0.044	0.00588±0.00068
Colera	25	14 (4)	9.433	0.883±0.052	0.00534±0.00069
Formentera	27	14 (4)	9.032	0.889±0.041	0.00511±0.00041
Cabrera	16	8 (3)	7.625	0.825±0.076	0.00493±0.00067
Scandola	21	10 (3)	8.199	0.886±0.043	0.00589±0.00069
Populonia	27	11 (1)	8.179	0.889±0.035	0.00529±0.00057
Crete	29	14 (4)	9.400	0.916±0.029	0.00492±0.00068
Kos	27	13 (5)	8.517	0.875±0.044	0.00503±0.00063
Rhodes	27	14 (7)	9.026	0.883±0.045	0.00550±0.00053
**Total Mediterranean**	**446**	**109 (94)**	**8.930**	**0.881±0.010**	**0.00519±0.00014**
**TOTAL**	**604**	**161**	**9.954**	**0.912±0.007**	**0.00658±0.00026**

*N*: sample size, *N_h_*: number of haplotypes, *N_priv_*: number of private haplotypes, *r_hap_*: haplotype richness after rarefaction to a sample size of 15, *H*: haplotype diversity, *π*: nucleotide diversity, *SD*: standard deviation.

The analysis of haplotype relationships using baps clustered the sampled haplotypes into three haplogroups (henceforth named A, B & C). Haplogroup A is the most abundant in all Eastern Atlantic and Mediterranean populations, but it is absent from Brazil, haplogroup B can be found in all three regions and haplogroup C is exclusive from Brazilian populations (Fig. 1).

### Haplotype Network and Phylogenetic Inference

**Figure 2:**
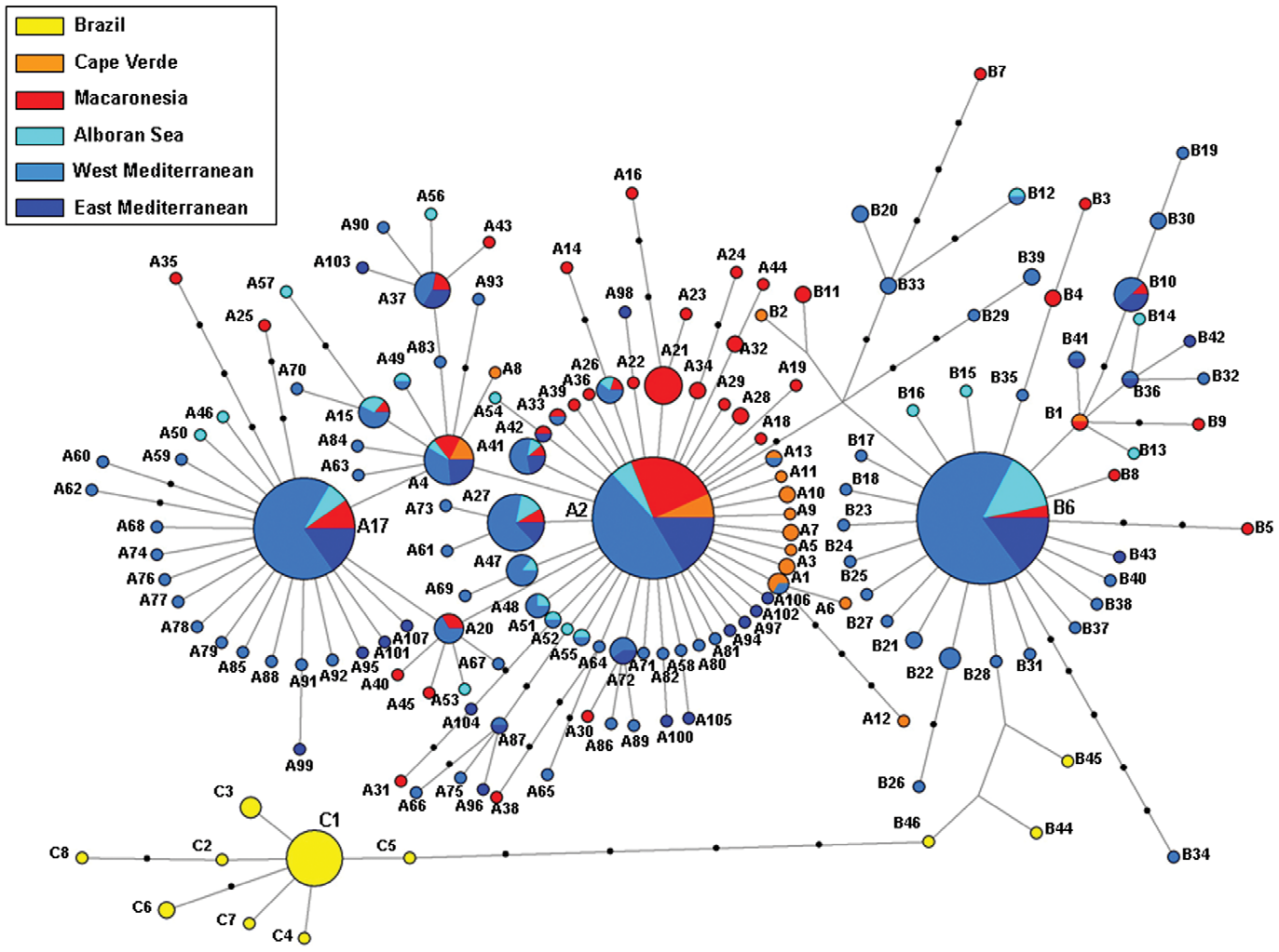
Median-joining haplotype network for *Arbacia lixula* COI. Haplotype numbers are preceded by a letter indicating the haplogroup they belong, A, B or C. Each haplotype is depicted by a circle coloured after the sub-region where it has been sampled. Areas are proportional to haplotype frequency. Each line represents a single nucleotide substitution step and additional mutations are represented by black bullets. The four haplotypes occupying central positions in each haplogroup, A2, A17, B6 and C1 are labelled in bigger font size.

The haplotype network (Fig. 2) showed a strikingly star-shaped topology with a high ratio of singletons (81.4% of all haplotypes), which is typical of populations that have suffered a recent demographic expansion. The three most abundant haplotypes (A2, A17, B6) occupy central positions. All initial loops obtained by the MP criterion could be resolved using coalescent theory, except one, comprising 2 of the most frequent haplotypes (A2, A17), plus haplotypes, A4 & A20, which is therefore left unresolved in the figure. The outgroup weights calculated by the tcs program identified A2 as the ancestral haplotype (Table S1). This is the second most frequent haplotype and the only which is present in all localities except in the Brazilian ones. Haplotypes of groups A & B, widely shared among Eastern Atlantic and Mediterranean populations, appear close together in the network. Conversely, the Brazilian private haplogroup C is separated by six mutation steps from haplogroup B. The three haplotypes belonging to group B that are present in Brazilian populations are the most closely related to haplogroup C.

**Figure 3:**
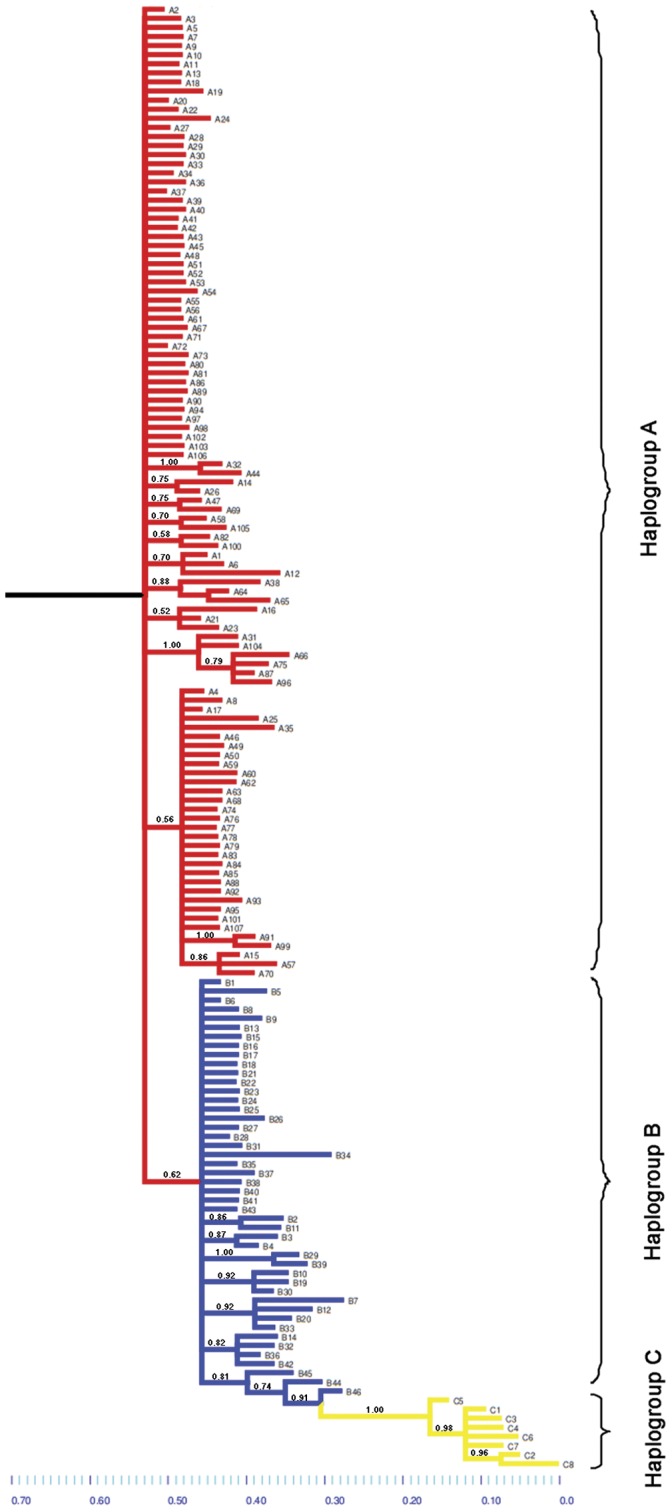
Bayesian inference consensus tree for haplotypes of *Arbacia lixula* COI. The tree is rooted using *Strongylocentrotus purpuratus* as outgroup (not shown); values for posterior probabilities >0.5, supporting non-collapsed clades, are indicated.

The consensus phylogenetic tree obtained by Bayesian Inference (Fig. 3) is coherent with the topology of the haplotype network. Haplotypes belonging to haplogroup A were collapsed at the base of the phylogram, indicating that this group is paraphyletic and ancestral, in accordance with the results of the outgroup weights analysis. Haplotypes of group B form a homogenous clade from which haplogroup C derives. The collapsed comb-like shape of haplogroups A and B suggests a recent demographic expansion. Interestingly, Brazilian haplotypes B44, B45 & B46 formed a monophyletic clade with haplogroup C, supported by a PP value of 0.81. This is consistent with previous results by Lessios *et al.* [26] which found that the samples from Brazil included in their analysis formed a clade nested within Eastern Atlantic (and Mediterranean) sequences.

### Population Structure

The analyses of population pairwise genetic differentiation (*F*_st_ and Jost’s *D*, Table 3) reflected a lack of population structure within both Eastern Atlantic and Mediterranean regions, but a clear differentiation between them and a complete differentiation (no alleles shared) of both regions from the Brazilian samples. Results from *F*_st_ and *D* were largely consistent. No significant differences could be found between any pair of localities from Cape Verde and Macaronesia, suggesting a high level of genetic flow among these Eastern Atlantic sub-regions. Likewise, no significant differences were found between any pair of Mediterranean localities (out of 136 possible pairs), with the exception of Torremuelle (the westernmost Mediterranean locality) where *F*_st_ analysis showed significant differences with two other Mediterranean localities, though these differences were not significant when *D* measures were analysed. Between Eastern Atlantic and Mediterranean, however, 38 (*D*) and 31 (*F*_st_) comparisons (out of 85) were significant. Remarkably, the localities of Carboneras (Western Mediterranean), Crete and Kos (Eastern Mediterranean) did not show any significant difference to any other Eastern Atlantic or Mediterranean population, despite the large geographical distances involved in the case of the two latter localities.

**Table 3:** Genetic differentiation between *Arbacia lixula* populations, *F*_st_ (below the diagonal) and Jost’s *D* (above the diagonal).

	Brazil		East Atlantic					Mediterranean																
	ITA	CFR	BOA	GIG	TEN	FAI	PIC	TOR	HER	CAR	PAL	VIL	BEN	XAB	CLM	TOS	COL	FOR	CAB	SCA	POP	CRE	KOS	ROD
**ITA**		0.115	**1** *	**1** *	**1** *	**1** *	**1** *	**1** *	**1** *	**1** *	**1** *	**1** *	**1** *	**1** *	**1** *	**1** *	**1** *	**1** *	**1** *	**1** *	**1** *	**1** *	**1** *	**1** *
**CFR**	0.132*		**1** *	**1** *	**1** *	**1** *	**1** *	**1** *	**1** *	**1** *	**1** *	**1** *	**1** *	**1** *	**1** *	**1** *	**1** *	**1** *	**1** *	**1** *	**1** *	**1** *	**1** *	**1** *
**BOA**	**0.876** *	**0.740** *		0.170	0.012	0.064	0.413	**0.671** *	0.763*	0.275	**0.553** *	**0.598** *	**0.557** *	**0.656** *	0.225	**0.889** *	**0.751** *	**0.664** *	0.510	**0.632** *	**0.608** *	0.181	0.270	**0.715** *
**GIG**	**0.865** *	**0.712** *	0.030		0.039	0.024	0.215	0.445	0.555*	0.129	0.343	0.380	0.290	0.427	0.093	0.720*	**0.669** *	0.512*	0.252	0.443	0.407	0.203	0.096	0.500*
**TEN**	**0.825** *	**0.676** *	0.015	0.002		0	0.037	**0.662** *	0.499*	0.216	0.431	0.524*	**0.454** *	0.453*	0.081	0.677*	0.448	0.453*	0.444	0.382	0.295	0.142	0.087	0.522*
**FAI**	**0.861** *	**0.719** *	0.007	0.022	0.000		0.038	**0.670** *	0.623*	0.226	**0.482** *	**0.531** *	**0.500** *	**0.559** *	0.185	**0.772** *	**0.608** *	**0.544** *	0.496	**0.510** *	**0.495** *	0.130	0.197	**0.605** *
**PIC**	**0.832** *	**0.679** *	0.035	0.010	−0.006	0.013		**0.501** *	0.150	0.214	0.226	0.309	0.272	0.178	0.081	0.230	0.097	0.102	0.326	0.092	0.067	0.124	0.040	0.146
**TOR**	**0.830** *	**0.674** *	**0.228** *	0.136*	**0.143** *	**0.222** *	**0.135** *		0.164	0.015	0	0	0	0.015	0.150	0.205	0.430	0.121	0	0.187	0.187	0.153	0.216	0.064
**HER**	**0.833** *	**0.687** *	0.053	0.043	0.002	0.043	0.001	0.113*		0.084	0	0	0.018	0	0.103	0	0	0	0.122	0	0	0.136	0.121	0
**CAR**	**0.846** *	**0.697** *	0.063	0.015	0.019	0.056	0.017	0.053	0.002		0	0	0	0	0	0.241	0.322	0.059	0	0.071	0.073	0	0	0.045
**PAL**	**0.820** *	**0.670** *	**0.101** *	0.047	0.039	**0.092** *	0.028	0.024	0.003	−0.014		0	0	0	0	0.008	0.118	0	0	0	0	0.020	0.013	0
**VIL**	**0.810** *	**0.662** *	**0.100** *	0.038	0.043	**0.095** *	0.036	0.015	0.013	−0.017	−0.020		0	0	0.037	0.074	0.227	0	0	0.019	0.042	0	0.110	0
**BEN**	**0.849** *	**0.701** *	**0.177** *	0.090	**0.097** *	**0.172** *	0.083	−0.010	0.061	0.011	−0.006	−0.012		0	0	0.083	0.231	0	0	0.010	0.039	0.082	0.015	0
**XAB**	**0.814** *	**0.659** *	**0.148** *	0.072	0.075	**0.142** *	0.061	−0.006	0.039	0.002	−0.012	−0.020	−0.020		0.030	0	0.089	0	0	0	0	0	0.074	0
**CLM**	**0.822** *	**0.667** *	0.093*	0.057	0.027	0.084*	0.019	0.051	−0.012	−0.002	−0.019	−0.011	0.015	0.003		0.224	0.201	0.022	0	0.011	0.022	0	0	0.045
**TOS**	**0.805** *	**0.659** *	**0.123** *	0.077	0.051	**0.115** *	0.035	0.028	0.008	0.005	−0.018	−0.007	−0.000	−0.007	−0.021		0	0	0.174	0	0	0.269	0.194	0
**COL**	**0.831** *	**0.682** *	**0.092** *	**0.084** *	0.025	**0.077** *	0.020	0.113*	−0.020	0.024	0.005	0.025	0.073	0.046	−0.017	0.001		0	0.338	0	0	0.236	0.175	0.009
**FOR**	**0.829** *	**0.681** *	**0.085** *	0.053	0.020	**0.074** *	0.013	0.065	−0.018	−0.009	−0.019	−0.006	0.024	0.010	−0.026	−0.017	−0.020		0.062	0	0	0.054	0.047	0
**CAB**	**0.858** *	**0.676** *	0.099*	0.037	0.026	0.088*	0.020	0.022	−0.001	−0.023	−0.028	−0.029	−0.015	−0.022	−0.029	−0.022	0.010	−0.023		0.051	0.093	0.069	0.001	0.003
**SCA**	**0.819** *	**0.651** *	**0.122** *	0.069	0.044	**0.111** *	0.026	0.037	0.002	0.002	−0.018	−0.007	0.006	−0.014	−0.022	−0.020	0.000	−0.017	−0.025		0	0.101	0	0
**POP**	**0.826** *	**0.680** *	**0.096** *	0.067	0.027	**0.087** *	0.018	0.063	−0.014	0.001	−0.014	0.000	0.028	0.011	−0.023	−0.021	−0.019	−0.020	−0.015	−0.017		0.105	0	0
**CRE**	**0.833** *	**0.693** *	0.037	0.016	0.003	0.030	0.004	0.088	−0.008	−0.011	0.001	0.002	0.047	0.027	−0.003	0.016	0.007	−0.005	−0.014	0.014	−0.001		0	0.105
**KOS**	**0.833** *	**0.686** *	0.064	0.025	0.013	0.058	0.005	0.053	−0.004	−0.016	−0.013	−0.008	0.013	0.007	−0.016	−0.011	0.008	−0.014	−0.029	−0.005	−0.016	−0.019		0.047
**ROD**	**0.817** *	**0.666** *	**0.100** *	0.055	0.034	**0.091** *	0.014	0.037	−0.004	−0.008	−0.021	−0.011	0.003	−0.009	−0.023	−0.024	−0.004	−0.023	−0.026	−0.024	−0.023	−0.000	−0.020	

Consistently significant differences obtained by both methods after false discovery rate correction are represented in bold.

Significant *P* values for *F*_st_ obtained from randomization. *: significant after false discovery rate correction (*P*<0.0085).

Significant *P* values for *D* indicate that confidence interval obtained by bootstrapping excludes 0.

* : significant after false discovery rate correction (*P*<0.0085).

**Figure 4:**
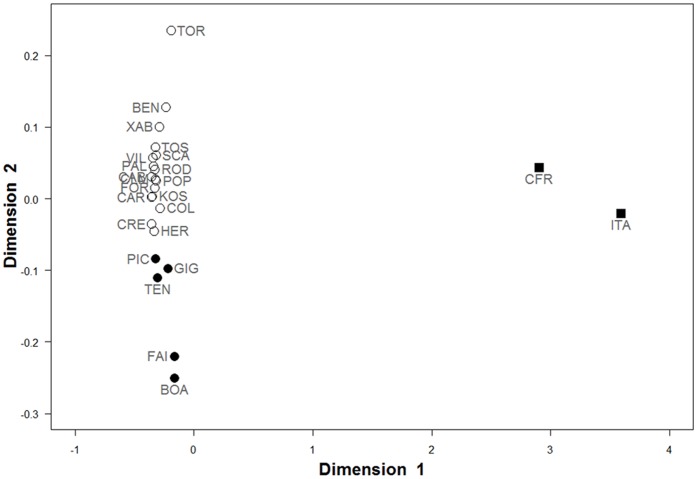
Multidimensional scaling (MDS) for *F*_st_ differentiation of *Arbacia lixula* COI haplotypes. Filled squares (▪) represent Brazilian populations, whereas filled circles (•) represent Eastern Atlantic populations and open circles (○) correspond to Mediterranean populations.

The MDS analysis (Fig. 4) graphically expresses the relationships among populations obtained from *F*_st_ measures. Brazilian localities are widely separated in the first dimension from Eastern Atlantic and Mediterranean populations, whereas the Mediterranean and Eastern Atlantic populations were separated along the second axis. The lack of structure between sub-regions within the Eastern Atlantic and the Mediterranean is also apparent in the graphical arrangement. The same analysis using *D* measures (not shown) reflected the same overall structure.

Consistent with the pairwise differentiation analysis, the AMOVA found significant differences between the three regions (Table 4), which remained significant when only Eastern Atlantic *vs.* Mediterranean regions were compared (Table 5). Conversely, and again in agreement with the pairwise differentiation analyses, no significant differences within regions between Eastern Atlantic sub-regions (Table 6) or among the three Mediterranean sub-basins (Table 7) were detected by AMOVA. The same results were obtained when these AMOVAs were repeated considering genetic distances between haplotypes (data not shown).

**Table 4:** Analysis of molecular variance (AMOVA) among regions using COI haplotype frequencies. Brazil vs. East Atlantic vs. Mediterranean.

Source of variation	df	Sum of squares	Variance components	Variation %	*P* value	Fixation index
Among groups	2	12.530	0.04690	9.69	<0.0001***	0.09692
Among populations within groups	21	9.728	0.00107	0.22	0.2583	0.00245
Within populations	580	252.833	0.43592	90.09	<0.0001***	0.09913
Total	603	275.091	0.48389			

**Table 5:** Analysis of molecular variance (AMOVA) among regions using COI haplotype frequencies. East Atlantic vs. Mediterranean.

Source of variation	df	Sum of squares	Variance components	Variation %	*P* value	Fixation index
Between groups	1	4.104	0.01893	4.08	<0.0001***	0.04081
Among populations within groups	20	9.075	0.00035	0.08	0.3916	0.00080
Within populations	547	243.200	0.44461	95.84	0.0002***	0.04157
Total	568	256.380	0.46389			

**Figure 5:**
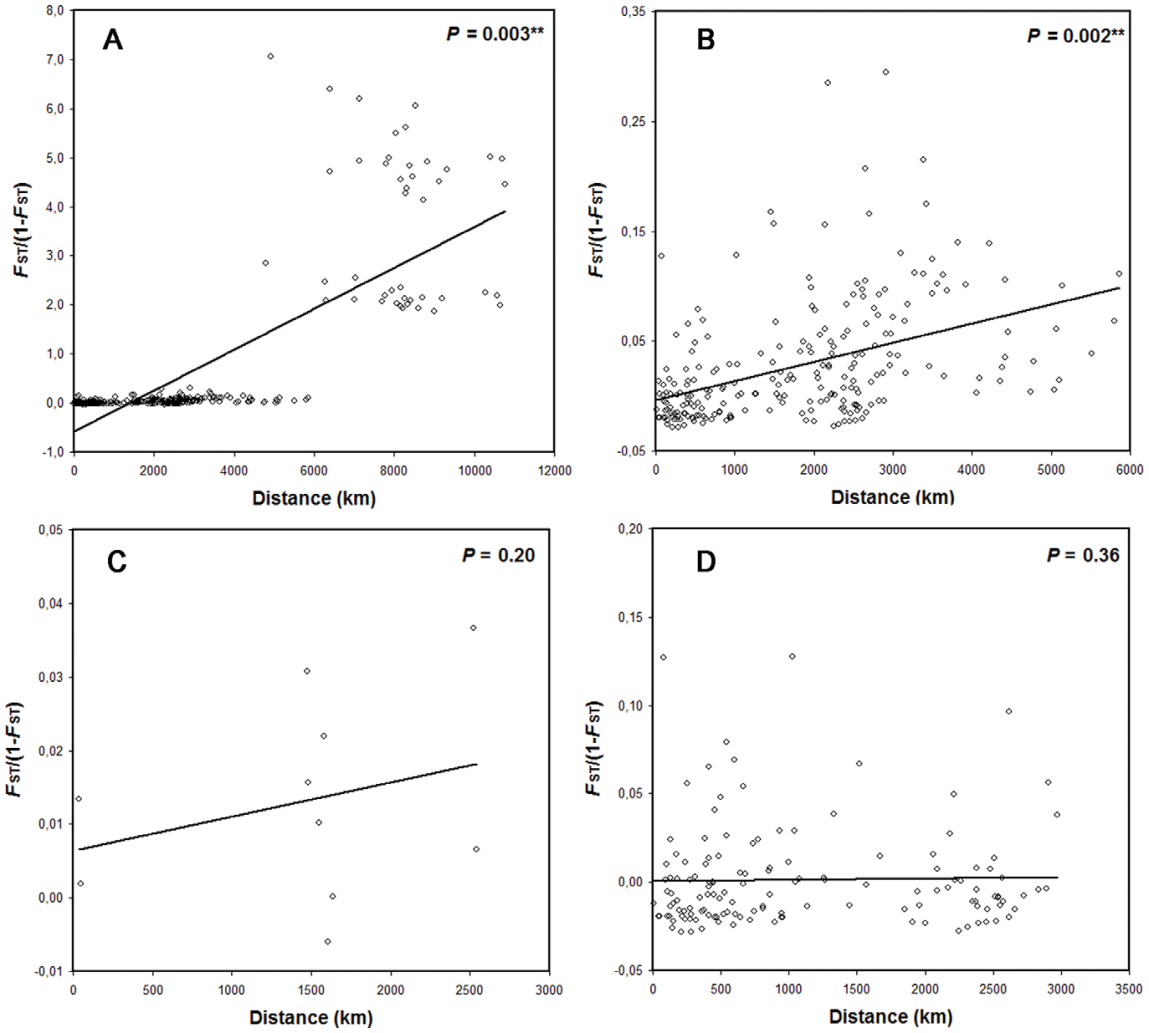
Relationships between genetic and geographic distances for different datasets of *Arbacia lixula* populations. Results of the Mantel test for isolation by distance are indicated.

The Mantel test showed significant isolation by distance when the whole dataset was analyzed (Fig. 5A). This result remained significant when populations from Brazil were excluded (Fig. 5B). Contrarily, no significant correlation between genetic differentiation and geographical distance was found when populations within just one region, either East Atlantic or Mediterranean, were analyzed (Fig. 5C & 5D).

**Table 6:** Analysis of molecular variance (AMOVA) among sub-regions within Eastern Atlantic region, using COI haplotype frequencies: Macaronesia vs. Cape Verde.

Source of variation	df	Sum of squares	Variance components	Variation %	*P* value	Fixation index
Among groups	1	0.681	0.00483	1.04	0.400	0.01042
Among populations within groups	3	1.427	0.00074	0.16	0.385	0.00161
Within populations	118	54.046	0.45802	98.80	0.179	0.01201
Total	122	56.154	0.46359			

**Table 7:** Analysis of molecular variance (AMOVA) among sub-regions within the Mediterranean, using COI haplotype frequencies: Alboran vs. Western Mediterranean vs. Eastern Mediterranean.

Source of variation	df	Sum of squares	Variance components	Variation %	*P* value	Fixation index
Between groups	2	0.829	−0.00023	−0.05	0.495	−0.00052
Among populations within groups	14	6.138	−0.00009	−0.02	0.482	−0.00021
Within populations	429	189.154	0.44092	100.07	0.514	−0.00073
Total	445	196.121	0.44059			

### Historical Demography

**Figure 6:**
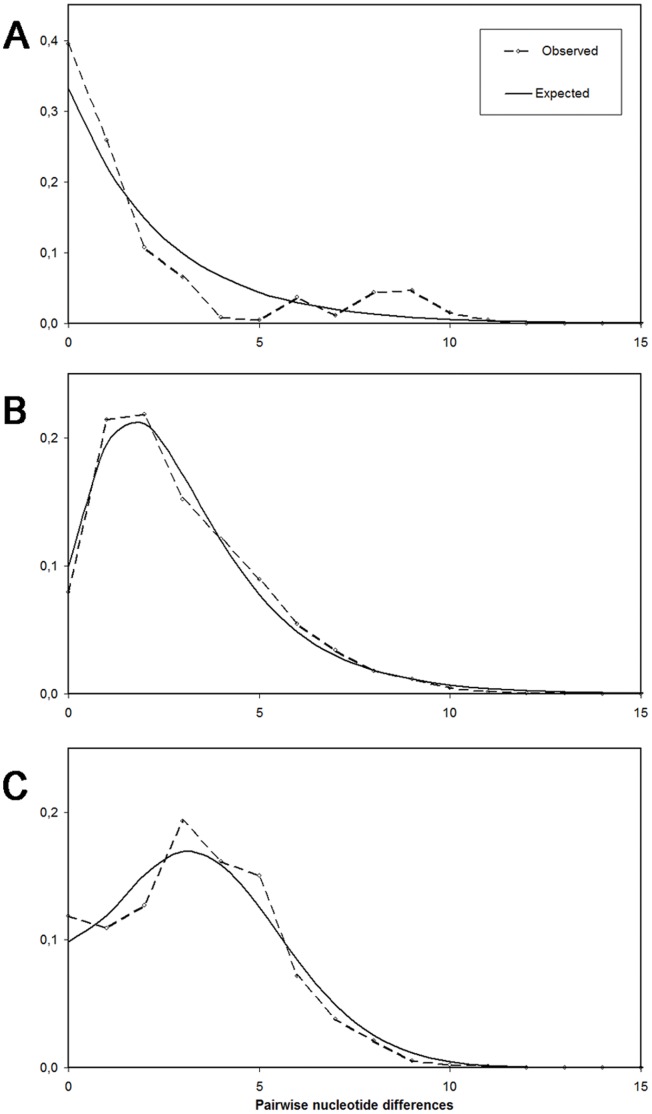
Mismatch distributions of *Arbacia lixula* populations in the three studied regions. Observed data and theoretical expected distributions are represented by discontinuous and solid lines, respectively. For Brazil (A), the theoretical expected distribution shown is that of a population of constant size. In the case of the East Atlantic (B) and the Mediterranean (C), data were fitted to a sudden expansion model.

The mismatch distribution of *Arbacia lixula* populations from the Brazilian region (Fig. 6A) did not fit the sudden expansion model (Table 8). Conversely, the mismatch distribution for the Eastern Atlantic region (Fig. 6B) was remarkably unimodal. This indicates that a recent demographic expansion has occurred in this population. Similar results were obtained when only the Macaronesian sub-region was analyzed (Table 8). However, the distribution for the Cape Verde sub-basin did not fit the sudden expansion model, as reflected by a high SSD (Table 8). Nevertheless, this result may be an artefact due to small sample size (n = 27). The demographic expansion in the Eastern Atlantic populations could be dated, from the value of τ and the known mutation rate for the COI of Echinoidea, between 30.6–66.9 kya (thousand years ago), which is a surprisingly recent time.

**Table 8:** Mismatch distribution parameters for *Arbacia lixula* populations.

Region	*SSD*	*τ*	*θ_0_*	*θ_1_*	Estimated expansion time (kya)
Brazil	0.3525 **	N.A.	N.A.	N.A.	N.A.
Cape Verde	0.0265 *	N.A.	N.A.	N.A.	N.A.
Macaronesia	0.0004 ns	1.39	1.850	1000	31.3–68.4
Pooled East Atlantic	0.0014 ns	1.36	1.286	1000	30.6–66.9
Alboran Sea	0.0067 ns	4.24	0.000	12.54	95.4–208.7
West Mediterranean	0.0028 ns	4.20	0.000	9.75	94.5–206.7
East Mediterranean	0.0024 ns	3.90	0.001	10.86	87.7–191.9
Pooled Mediterranean	0.0026 ns	4.17	0.000	10.20	93.8–205.2
Whole Dataset	0.0030 ns	2.91	1.376	13.13	65.5–143.2

*SSD* values and their significances are presented along with sudden expansion model parameters and estimated time for the expansion (where applicable), for the studied regions and sub-regions and for the whole dataset.

* : Significant at *P*<0.05.

** : Significant at *P*<0.01.

ns : Not significant.

N.A.: Not applicable (sudden expansion model rejected).

The mismatch distribution obtained for the Mediterranean region (Fig. 6C) was also typically unimodal. The parameters of the theoretical curves calculated individually for each Mediterranean sub-basin had all similar values, comparable to those of the whole Mediterranean region (Table 8), reinforcing the idea that all the Mediterranean *Arbacia lixula* populations belong to the same genetic pool. The demographic expansion in the Mediterranean could be dated between 93.8–205.2 kya. This estimation is a little older than that obtained for the Eastern Atlantic expansion, but is still a recent time.

The neutrality and population expansion tests calculated for the different regions and sub-basins (Table 9) were largely coherent with the results inferred from the mismatch distributions. Tajima’s *D* detected significant differences from neutrality in all cases, except for Brazil and the Eastern Mediterranean sub-basin. Fu’s *F*_s_ test for demographic expansion was significant in all cases (though just marginally so in the case of Brazil). Ramos-Onsins & Rozas’ *R*_2_ was significant for all cases except the Eastern Mediterranean sub-basin, and the raggedness value *r* was consistent with unimodal distributions, except for Brazilian and Cape Verdean populations.

**Table 9:** Neutrality and population expansion tests for *Arbacia lixula* in the studied regions or sub-regions and for the whole dataset.

Region	*N*	*D*	*F_s_*	*R*_2_	*r*
Brazil	35	−1.80405 ns	−3.712 *	0.0566 **	0.0503 *
Cape Verde	27	−2.08319 *	−9.809 ***	0.0527 ***	0.1254 *
Macaronesia	96	−2.40571 **	−45.988 ***	0.0234 ***	0.0167 ns
Pooled East Atlantic	123	−2.51677 ***	−70.825 ***	0.0185 ***	0.0265 ns
Alboran Sea	53	−1.83549 *	−15.648 ***	0.0421 **	0.0221 ns
West Mediterranean	310	−2.25417 **	−98.101 ***	0.0187 **	0.0137 ns
East Mediterranean	83	−1.49411 ns	−17.677 ***	0.0494 ns	0.0160 ns
Pooled Mediterranean	446	−2.28043 **	−155.806 ***	0.0162 ***	0.0137 ns
Whole Dataset	604	−2.32451 **	−256.026 ***	0.0150 **	0.0094 ns

Tajima’s *D*, Fu’s *F_s_* statistic, Ramos-Onsins & Rozas’ statistic (*R*_2_), and raggedness index (*r*).

* : Significant at *P*<0.05;

** : Significant at *P*<0.01;

*** : Significant at *P*<0.001;

ns : Not significant.

## Discussion

COI and other mitochondrial markers have proven to be the most useful tool for tracing both intraspecific and intrageneric genealogies of many echinoid species [26], [64]–[65], [70]–[73] and usually yield easily interpretable results which are consistent with those of other nuclear markers. Nevertheless, our analyses are based on a single mitochondrial marker (COI). Thus, these results must be taken with caution, and further analyses using nuclear markers would be desirable. On the other hand, previous works in Echinoidea have shown that other nuclear markers were mainly used only to confirm the evolutionary history depicted by mtDNA [26], [72] or else displayed too much diversity to produce interpretable results [73].

The *Arbacia lixula* populations sampled showed high values of haplotype diversity and haplotype richness, but relatively low values of nucleotide diversity. The lowest diversity was found in Brazilian populations and, specifically, in the westernmost locality (Itaipu), which is close to the distribution limit of the species and separated from the other Brazilian locality by the Cabo Frio upwelling. In contrast, the highest diversity was found in the East Atlantic, as expected if this region is the geographical origin of the species [16], [26]. We detected three haplogroups in *A. lixula*. One of them (Group A) seems to be ancestral and is found only in Eastern Atlantic and Mediterranean populations, while another (Group B) is present at both sides of the Atlantic. The third one (Group C) is derived from Group B and found only in Brazil.

In a recent work, Lessios *et al.* [26] concluded that *Arbacia lixula* split from a common ancestor with *A. punctulata* ca. 2.6 Mya, and attributed this split to the mid-Atlantic barrier, separating the western *A. punctulata* from the eastern *A. lixula*, which would later have crossed back this barrier to establish itself, as an isolated clade, in the coast of Brazil. A problem with this view is that the mid-Atlantic barrier was fully in place long before the estimated date of the split, so the separation of the two species could not be a vicariance event but a range expansion event (on the part of the lineage that would become *A. lixula*), and two crossings of the barrier are required to fully explain the present-day distribution of the species (though the second crossing could be facilitated by the South Equatorial Current system [74]). An alternative scenario would be that the two Atlantic species diverged in Western Atlantic, after the rise of the Panama isthmus isolated their ancestor from the eastern Pacific region (the possible origin of the genus *Arbacia* [26]), and that *A. lixula* crossed the Atlantic ridge only once to colonize the Eastern Atlantic. Our results favour the first (Lessios’) view, as the haplotypes from Brazil formed a derived monophyletic group nested within the amphi-Atlantic Group B, rather than the opposite. This indicates a derived lineage in Western Atlantic, old enough to have had time to evolve forming the haplotype Group C. A more thorough sampling of the whole range of the Western Atlantic distribution and the inclusion of more data from Western Africa, are necessary before firm evidence can be obtained about the historical whereabouts of the main lineages of *A. lixula*.

Overall, the pattern of distribution of genetic variability (as shown in *F*_st_, Jost’s *D*, MDS and AMOVA analyses) showed three groups of populations that differed significantly from each other (Brazilian, Eastern Atlantic and Mediterranean), while little structure could be found within these groups. It is remarkable that the *F*_st_ measures based on sequence distance metrics and the differentiation measure *D* based on haplotype frequencies yielded essentially the same results. This is attributable to the prevalence of close haplotypes separated by small number of mutations (hence the low nucleotide diversity in general) that are widespread among populations. Thus, haplotype genetic differences had relatively little weight and most population structure derives from haplotype frequency differences.

Another striking pattern resulting from our molecular analyses is that recent demographic phenomena have shaped the present-day genetic structure of *Arbacia lixula* populations in the Eastern Atlantic and the Mediterranean. This does not seem to be the case of the Brazilian population but, given the small sample size, it is unclear if the resulting mismatch distribution (Fig. 6A) is either multimodal or L-shaped in this population. Multimodal curves are typical of populations at demographic equilibrium, but L-shaped distributions may result from very recent demographic bottlenecks [75]. More extensive sampling would be required to get the full picture of the demographic processes that have shaped the Brazilian populations of *A. lixula*.

The lack of an exclusively Mediterranean mitochondrial lineage of *Arbacia lixula* is remarkable. Other Atlanto-Mediterranean echinoderms such as *Marthasterias glacialis* [76], *Holothuria mammata* [77] or *Paracentrotus lividus* [73], [78] do have lineages exclusive of the Mediterranean. These species have been probably present in the Mediterranean for several million years and their populations may have suffered several episodes of impaired gene flow during the Pleistocene glaciations. The genetic structure shown by *A. lixula* probably reflects a different demographic history from these other species.

Even if there is no phylogenetic break in the Mediterranean (as also found by Lessios *et al.* [26]) and alleles are widely shared at both sides of the Gibraltar boundary, this barrier seems nevertheless to restrict gene flow in *Arbacia lixula*, so as to establish significant differences in terms of haplotype frequencies between Mediterranean and Eastern Atlantic populations. The AMOVA (and pairwise comparisons) detected significant genetic differentiation between these groups of populations (Table 5), suggesting a reduced gene flow through the Strait of Gibraltar. Differently to what can be found in other marine organisms [79], the Strait itself, and not the Almeria-Oran Front (some 350 Km east of Gibraltar), is the place of the phylogeographic break, as the populations from the Alboran Sea are undistinguishable from other Mediterranean populations, but are significantly differentiated from most Atlantic populations (Fig. 4, Tables 3 and 7). Thus, *A. lixula* does not show any genetic differentiation among populations throughout the whole Mediterranean Sea. This could be due to recurrent gene flow, but oceanographic barriers such as the Almeria-Oran Front or the Siculo-Tunisian Strait [79] are strong enough to maintain genetic differentiation among different sub-basins in the case of other echinoderms of similar larval dispersive capacity [73], [77]–[78]. We favour the alternative explanation (for the lack of genetic structure) that the colonization of the Mediterranean by *A. lixula* is so recent (see below) that populations in the different Mediterranean sub-basins have not had yet enough time to diverge from each other.

In the case of Macaronesian and Cape Verdean populations (Table 6), it seems likely that the present-day genetic similarity could be the result of a recent demographic expansion (see below), which could have swamped any trace of previous differentiated lineages potentially formed during periods of restricted gene flow among archipelagos.

Brazilian populations of *Arbacia lixula* are completely differentiated from Eastern Atlantic and Mediterranean populations (Tables 3 & 4). In addition, they showed the lowest genetic diversity and did not show any signature of demographic expansion. Nevertheless, our sample size is small, and Northern and Central Brazilian populations of *A. lixula* have never been sampled for phylogeographic studies. More extensive sampling along the Brazilian coast would be required for a full understanding of factors shaping the genetic structure of the West Atlantic populations of *A. lixula*.

The almost complete lack of fossil record for *Arbacia lixula* in the Mediterranean is most revealing. At present, the species is highly abundant and occurs in areas that have been thoroughly sampled by palaeontologists. Other Mediterranean echinoids currently co-occurring in the same habitats are commonly found in assemblages of the Pleistocene and have been abundantly reported in the paleontological literature [80]–[83]. In contrast, only one fossil individual of *A. lixula* from the Mediterranean has ever been reported in the literature [13]. It was found in very young deposits from Livorno (Italy) whose recency led Stefanini to speculate that *A. lixula* had an exotic origin and had entered the Mediterranean in recent times [13]. *A. lixula* is consistently absent from fossil assemblages of the so-called “Senegalese fauna” that characterize the warmer periods from the Tyrrhenian stage (ca. 260–11.4 kya), which have been extensively sampled and thoroughly described [84]–[89].

As for the Atlantic archipelagos, recent work on the fossil echinoid fauna of Azores Islands [90] has revealed the presence of *A. lixula*, providing several tens of pieces of individuals, including the oldest known record of this species. These deposits are currently dated to 130–120 kya [91], which corresponds to the last interglacial or Riss-Würm (also called MIS 5e, ca. 130–114 kya). These specimens add up to the only other Atlantic *A. lixula* fossil specimen known from the Pleistocene of Madeira [13] whose dating is more uncertain.

Thus, there is scarce paleontological evidence of the occurrence of *Arbacia lixula* in the Mediterranean, and somewhat more, but still scarce, evidence of the colonization of the Atlantic archipelagos of Azores and Madeira, which probably occurred during the last interglacial period of the Pleistocene (MIS 5e). These observations are in agreement with the genetic signatures we observed in the mismatch distributions, which clearly show that recent sudden expansions have occurred in the Mediterranean and Macaronesian populations (Fig. 6). This is also supported by the strikingly star-shaped topologies of the haplotype network (Fig. 2) and by the comb-like clades in the BI phylogenetic tree (Fig. 3). Our temporal estimation for the demographic expansion in the Mediterranean (93.8–205.2 kya) is coherent with the only available fossil record [13]. This is considerably younger than the times for expansion events found in other Mediterranean echinoderms using the same estimation method, which vary from 300 to 600 kya [73], [77] and fits with the possibility that the colonization of the Mediterranean by *A. lixula* took place as recently as during the last interglacial period (MIS 5e). This period was also the longest of all interglacial warm periods of the Pleistocene. The minimum winter surface temperature of the Mediterranean Sea stayed warmer than 19°C for several thousands of years [89]. This probably enabled tropical Atlantic populations of *A. lixula* to cross the Strait of Gibraltar and colonize the Mediterranean.

In the case of Eastern Atlantic populations, the exponential demographic expansion is even more apparent, since the mismatch distribution follows a sharp unimodal curve which fits to a sudden expansion model with a very high value for θ_1_. This expansion probably occurred more recently than in the Mediterranean (31.3–68.4 kya). This estimation falls within the Late Pleistocene, an epoch generally dominated by the last glaciation (Würm), during which the mean sea level dropped down to 80 m below the present level [92]–[93]. Changes in ocean circulation related to this sea level drop can be related to the population expansion of *A. lixula* in the Eastern Atlantic. Contrary to what happens in the Mediterranean, the fossils available show that the species was present in Macaronesia before this expansion [90], so the demographic history of the Atlantic populations of *A. lixula* seems to be more complex than that of the Mediterranean populations. To complete the picture of the colonization of Atlantic archipelagos, data from continental African shores would be highly valuable.

An invasive species can be defined as a “species that threatens the diversity or abundance of native species, the ecological stability of infested ecosystems, economic activities (e.g., agricultural, aquacultural, commercial, or recreational) dependent on these ecosystems and/or human health” [94]. Although the term is generally applied to species introduced as a result of human activities, it should not be necessarily so. Moreover, ecosystem engineer species such as *Arbacia lixula*, that have shaped contemporary communities as the result of a colonization event that took place many years ago, can be falsely viewed as native [95]. According to our molecular data, *A. lixula* has indeed colonized the Mediterranean recently and complies with the terms of the former definition, even if it is usually viewed as native because its colonization took place following natural climatic changes, without human intervention.

Whether considered as an “old natural invader” or as native, the present trend of global warming can potentially boost the negative impact of *A. lixula* in Mediterranean ecosystems, thus possibly turning a “natural” colonization into an ecological problem related (at least partially) to human intervention. The ongoing warming [96] may facilitate population blooms of *A. lixula* in Northern Mediterranean, by releasing the constraint to larval development due to low water temperature. Warnings have been issued about its potential population increase and the generation of barren grounds in sublittoral habitats [10]–[11].

Thus, genetic data are in agreement with the consideration of *Arbacia lixula* as a thermophilous species that has recently colonised the Mediterranean and whose densities may increase in the foreseeable future. Monitoring of populations seems highly recommendable as a management tool in the near future for protecting the threatened Mediterranean shallow water ecosystems.

## Supporting Information

Table S1 Haplotype frequencies of *Arbacia lixula* COI for all sampled localities. Haplotypes shared by two or more localities are represented in **bold**, while numbers not in bold correspond to private haplotypes. Background colours correspond to the three different haplogroups. Outgroups weights calculated by tcs are also displayed for each haplotype, and that with the highest outgroup weight (A2) is highlighted in green background.
(XLS)
